# Gastroprotective Mechanisms of the Monoterpene 1,8-Cineole (Eucalyptol)

**DOI:** 10.1371/journal.pone.0134558

**Published:** 2015-08-05

**Authors:** Germana Freire Rocha Caldas, Alisson Rodrigo da Silva Oliveira, Alice Valença Araújo, Simone Sette Lopes Lafayette, Giwellington Silva Albuquerque, Jacinto da Costa Silva-Neto, João Henrique Costa-Silva, Fabiano Ferreira, José Galberto Martins da Costa, Almir Gonçalves Wanderley

**Affiliations:** 1 Department of Pharmaceutical Sciences, Universidade Federal de Pernambuco, 50740–521, Recife, PE, Brazil; 2 Department of Physiology and Pharmacology, Universidade Federal de Pernambuco, 50670–901, Recife, PE, Brazil; 3 Department of Histology and Embryology, Universidade Federal de Pernambuco, 50670–901, Recife, PE, Brazil; 4 Department of Physical Education and Sport Sciences, Universidade Federal de Pernambuco, 55608–680, Vitória de Santo Antão, PE, Brazil; 5 Department of Biological Chemistry, Universidade Regional do Cariri, 63105–000, Crato, CE, Brazil; IDIBAPS—Hospital Clinic de Barcelona, SPAIN

## Abstract

Recently, our research group identified and reported 1,8-cineole (CIN), a monoterpene that naturally occur in many aromatic plants, as one of the major constituent of the essential oil from leaves of *Hyptis martiusii* (EOHM), as well as characterized the gastroprotective action of this oil. The aim of this study was to investigate the mechanisms of action involved in the antiulcer and healing activity of CIN, in order to confirm its correlation with the gastroprotective effect of EOHM. Wistar rats were exposed to different protocols (acute ulceration, gastrointestinal motility and antisecretory activity). In addition, were determinated the involvement of nitric oxide and sulphydryl groups; the levels of gastric mucus, lipid peroxidation, sulphydryl groups and myeloperoxidase activity. The healing ability was evaluated by acetic acid-induced chronic ulcer and histological and immunohistochemical analysis (PCNA, Ki-67 and BrdU). The treatment with CIN inhibited ethanol-, ethanol/HCl- and indomethacin-induced gastric lesions. The highest doses of CIN inhibited gastric emptying, but did not affect intestinal transit. CIN (100 mg/kg) reduced the volume of basal but not stimulated acid secretion. CIN increased levels of mucus (89.3%), prevented depletion of –SH groups (62.6%) and reduced the level of lipid peroxidation (55.3%) and myeloperoxidase activity (59.4%) in the gastric mucosa. In chronic ulcer model, CIN reduced in 43.1% the gastric area lesion, promoted significant regeneration and restoration of the levels of mucus in glandular cells as confirmed by histological analysis; and promoted increase in cell proliferation as evidenced by reactivity for PCNA, Ki-67 and BrdU. This findings demonstrate the role of 1,8-cineole as an important ulcer healing agent and indicate the involvement of antioxidant and cytoprotective mechanisms in the gastroprotective effect of compound. This study also provides evidence that 1,8-cineole is related to the gastroprotective effect of the essential oil of *Hyptis martiusii*.

## Introduction

A wide variety of chemical substances, mixtures of herbs and plant extracts have been proved to possess therapeutic properties in experimental models of peptic ulcer. The gastroprotective effect of these compounds and extracts (combined or in isolation) has been attributed to three main functions, including antisecretory, cytoprotective and antioxidant properties [1].

1.8-cineole, also known as eucalyptol, is a monoterpene found naturally in many aromatic plants of the *Eucalyptus*, *Croton*, *Hyptis*, *Pectis*, *Rosamarinus and Salvia* genera [2, 3, 4]. Previous studies show that 1,8-cineole has been examined for a number of biological and pharmacology activities, including insecticidal and antimicrobial [5], antiallergic and anti-inflammatory [6], hepatoprotective [7], antitumoral [8] and gastroprotective activities [9].

In the specific case of the Hyptis genus, 1,8-cineole is reported to be the main compound in species such as *H*. *fruticosa*, *H*. *goyazensis*, *H*. *suaveolens* and *H*. *martiusii* [10]. *Hyptis martiusii* Benth. (Lamiaceae), popularly known as *cidreira-do-mato*, is characterized as a potential source of essential oils, like other species of the genus. A study conducted by Araújo et al. [2] showed that the essential oil of fresh leaves of *H*. *martiusii* consists of mono- and sesquiterpenes, and its major components are 1,8-cineole, δ-3-carene, bicyclogermacrene and β-caryophyllene.

In a study recently reported by our group [11] showed that the essential oil of *H*. *martiusii* (EOHM) has gastroprotective effect in various gastric lesion models in rats and that the main constituent of this oil is 1,8-cineole. The gastroprotective effect of EOHM involves both an antisecretory activity mediated by the histamine H_2_ and gastrin CCK_2_ receptors, as involves the participation of endogenous sulfhydryl groups, with an increase in basal levels of these groups, increasing the mucus secretion, reducing levels of lipid peroxidation and also accelerates the healing of chronic ulcers promoting significant regeneration of the gastric mucosa. As it has been previously described in the literature that 1,8-cineole inhibits ethanol-induced gastric lesions [9], we conducted a detailed investigation to check if 1,8-cineole was the responsible for the gastroprotective effect of EOHM and to evaluate the mechanisms of action involved in the antiulcer activity and ulcer healing properties of this compound.

## Material and Methods

### Reagents and Chemicals

The following substances were used: 1,8-cineole, sodium acetate, Alcian Blue, atropine, thiobarbituric acid, trichloroacetic acid, 5,5’-dithiobis (2-nitrobenzoic acid), bethanechol, carbenoxolone, EDTA, glutathione, histamine, N-acetylcysteine, N-ethylmaleimide, nitro_ω_-L-arginine methyl ester, pantoprazole, pentagastrin, ranitidine, sodium lauryl sulfate, 1,1,3,3-tetramethoxypropane, hexadecyltrimethylammonium bromide, 3,3´,5,5´-tetramethylbenzidine, dimethylformamide (Sigma-Aldrich, St. Louis, USA), tris (hydroxymethyl) aminomethane, acetic acid, hydrochloric acid, ethanol, n-butanol, magnesium chloride, sodium chloride, potassium chloride, glucose, sodium hydroxide, anhydrous sodium sulfate, polysorbate 80—Tween 80, hydrogen peroxide solution (Vetec, Duque de Caxias, Brazil), ethyl ether, formaldehyde, phenolphthalein (FMaia, Cotia, Brazil), xylazine, ketamine (Vetbrands, Paulinia, Brazil), PCNA antibody [PC10]—mouse monoclonal antibody (Abcan Inc., Cambridge, US), Ki-67 protein (code: sc-23900, Santa Cruz Biotechnology, Santa Cruz, CA, USA) and BrdU protein (code: sc-32323, Santa Cruz Biotechnology, Santa Cruz, CA, USA). For the purposes of the experiment, the 1,8-cineole (CIN) was emulsified in a Tween 80 at 1% before administration to the animals.

### Animals

Male and female Wistar rats weighing 200–300 g were obtained from the Department of Physiology and Pharmacology from Federal University of Pernambuco, Pernambuco, Brazil. They were kept under standard environmental conditions (12 h dark/light cycle) and temperature (22 ± 2°C). Water and industrialized dry food (Presence, Purina, Brazil) were made available *ad libitum*. All the experiments were conducted in accordance with the National Institute of Health’s Guide for the Care and Use of Laboratory Animals and were submitted to and approved by the Animal Experimentation Ethics Committee of the Federal University of Pernambuco (UFPE), under the license n°. 037544. In all protocols, the animals were euthanized in a CO_2_ chamber.

### Experimental assays

The 1,8-cineole (CIN) was emulsified in a 1% Tween-80 before administration to the animals. For each experimental model, the animals were randomly divided into three groups. The negative control group received a 1% Tween-80 aqueous solution; the positive control group received pantoprazole (a proton pump inhibitor), ranitidine (an antagonist of the histamine H_2_ receptor), atropine (a cholinergic antagonist), carbenoxolone (a cytoprotective agent) or N-acetylcysteine (a standard antioxidant drug) depending on the experimental model; and the treated groups received 1,8-cineole (CIN) at the doses of 50, 100 and 200 mg/kg in acute models of induction of gastric ulcer. The dose of 100 mg/kg of CIN was chosen for the additional studies in order to shed light into the mechanisms underlying its gastroprotective effect, as this had been shown to be the most effective dose in previously assessed protocols. Prior to each experiment, the animals were fasted because the treatments were orally administered, and they were kept in cages with raised floors of wide mesh to prevent coprophagy.

### Antiulcerogenic activity

#### Ethanol-induced gastric ulcer

After 16 h of fasting, the rats (n = 6/group, three females and three males) were orally treated with 1% Tween-80 aqueous solution (control), pantoprazole (40 mg/kg) or CIN (50, 100 and 200 mg/kg), 1 h before administration of the ulcerogenic agent. Gastric lesions were induced by using ethanol (70%, 0.5 mL/100 g) by oral route according to the method described by Robert et al. [12]. The animals were euthanized with CO_2_ gas 1 h after the induction of gastric lesions. The stomachs were removed, photographed and the area of gastric lesion determined by computerized planimetry (Software ImageJ). The data was expressed in mm^2^.

#### HCl/ethanol-induced ulcer

After 24 h of fasting, the rats (n = 6/group, three females and three males) were orally treated with 1% Tween-80 aqueous solution (control), pantoprazole (40 mg/kg) or CIN (50, 100 and 200 mg/kg). 1 h after treatment, all the animals received 0.3 M HCl/etanol 60% solution (1 mL/150 g) by oral route to induce acute gastric lesions [13]. The animals were euthanized with CO_2_ gas 1 h after the induction of gastric lesions. The stomachs were removed and examined for quantification of the lesions as described above.

#### Nonsteroidal anti-inflammatory drug (NSAID)-induced gastric ulcer

After 16 h of fasting, the rats (n = 6/group, three females and three males) were orally treated with 1% Tween-80 aqueous solution (control), pantoprazole (40 mg/kg) or CIN (50, 100 and 200 mg/kg). 30 min after the treatment, indomethacin (30 mg/kg) was administered subcutaneously to induce gastric lesions [14]. Six hours after the administration of indomethacin, the animals were euthanized with CO_2_ gas, the stomachs removed and inspected to determine the gastric lesions produced. The results were expressed as lesions, ulcers and total index, which were obtained from scores determined by various alterations in the gastric mucosa and the number and size of necro-hemorrhagic lesions [15].

### Effect of 1,8-cineole on gastrointestinal motility

#### Gastric emptying assay

After 6 h of fasting, the rats (n = 6/group, three females and three males) were orally treated with 1% Tween-80 aqueous solution (control) or CIN (50, 100 and 200 mg/kg), and subcutaneously with atropine (3 mg/kg). After 1 h or 30 min of the treatments, each animal received by oral route 1.5 mL of phenol red (0.5 mg/mL). The zero time control group was euthanized with CO_2_ gas immediately after the administration of the marker and the other groups were euthanized 30 min later. The stomachs were removed, the gastric content was collected and centrifuged at 176 × g for 15 min. After centrifugation, 1 mL aliquots of supernatants were added to 1 mL of 1 N NaOH. The absorbance of the solution was read at 560 nm and the results were expressed as the concentration (μg) of dye retained in the stomach in relation to the control group [16].

#### Intestinal transit assay

After removal of the rats’ stomachs in the gastric emptying model, the small intestine was removed for evaluation of intestinal transit. With the aid of a ruler, the total length of the small intestine of each animal and the distance traveled by the phenol red (until the last portion of the intestine containing at least 1 cm of continuous marker) was measured. The results were expressed as a percentage of the distance traveled by the marker in relation to the total length of the small intestine [17].

### Evaluation of mucosal protective factors

#### Determination of stimulated gastric acid secretion

The experiment was carried out using the pyloric ligature method described by Shay et al. [18], with slight modifications. The animals were divided into 11 groups (n = 6/group, three females and three males): (1) control, (2) CIN, (3) pantoprazole, (4) histamine, (5) histamine plus ranitidine, (6) histamine plus CIN, (7) bethanechol, (8) bethanechol plus atropine, (9) bethanechol plus CIN, (10) pentagastrin or (11) pentagastrin plus CIN. They were fasted for 16 h with free access to 5% glucose solution. For pyloric ligature, the animals were anaesthetized (xylazine, 6 mg/kg and ketamine, 60 mg/kg, intraperitoneally) and, immediately after the ligature, they received an intraduodenal dose of 1% Tween-80 aqueous solution (control, 0.1 mL/100 g body weight), ranitidine (60 mg/kg) or CIN (100 mg/kg) or subcutaneous atropine (1 mg/kg). The abdominal wall was sutured and, 1 h after pylorus ligation, the animals received subcutaneously a histamine (20 mg/kg), bethanechol (2.5 mg/kg) or pentagastrin (400 μg/kg) stimulus. Four hours after pylorus ligation, the animals were euthanized with CO_2_ gas. The gastric secretion was collected and centrifuged at 176 × g for 30 min. The volume (mL), pH values and the total acidity (mEquiv.[H+]/mL/4h) were determined.

#### Involvement of nitric oxide (NO) and sulfhydryl compounds (–SH groups) in gastroprotection

Rats fasted for 24 h were distributed into nine groups (n = 6/group three females and three males). Three groups were pretreated with 0.9% NaCl solution (intraperitoneally), three groups with L-NAME (N_ω_-nitro-L-arginine methyl ester, 70 mg/kg, intraperitoneally), an inhibitor of the NO-synthase enzyme and three groups with NEM (N-ethylmaleimide, 10 mg/kg, intraperitoneally), a sulfhydryl compound blocker [19, 20] to investigate the influence of endogenous NO and–SH groups on the gastroprotective effect of CIN. 30 min after the pretreatment, it was administered a 1% Tween-80 aqueous solution, carbenoxolone (100 mg/kg) or CIN (100 mg/kg), by oral route. One hour later, all the animals received 1 mL of absolute ethanol by oral route to induce gastric lesions. One hour after the administration of ethanol, the animals were euthanized with CO_2_ gas and their stomachs were removed for the determination of gastric lesions as previously described.

#### Quantification of gastric mucus

Quantification of gastric mucus was determined according to the methodology described by Corne et al. [21]. After a 16 h fasting, the animals (n = 6/group, 3 females and 3 males) were treated with 1% Tween-80 aqueous solution (CL), pantoprazole (40 mg/kg) or CIN (100 mg/kg). 1 h later, it was induced the gastric lesions by ethanol (70%, 0.5 mL/100 g, oral route). The non-injured control group (CN) received no treatment. The animals were euthanized 1 h later and their stomachs were removed. The glandular segment was weighed and transferred to a tube containing 10 mL of 0.1% Alcian Blue and stained for 2 h. The dye complexed to the mucus gland wall was extracted with 10 mL of magnesium chloride (0.5 mol/L) and agitated for 2 h. At 4 mL of the mixture, 4 mL of diethyl ether were added and the solution was shaken. The emulsion obtained was centrifuged at 1,480 × g for 10 min. The absorbance of samples was read at 598 nm and results were expressed as μg of Alcian Blue/g of tissue.

### *In vivo* antioxidant activity

The antioxidant tests were performed with the homogenate of the gastric mucosa of animals with ethanol-induced ulcers [12]. After fasting for 16 h, the animals were divided into four groups (n = 6/group, 3 females and 3 males) and treated orally with 1% Tween-80 aqueous solution (CL, injured control), N-acetylcysteine (NAC, 750 mg/kg) or CIN (100 mg/kg) 1 h before the administration of the ulcerogenic agent. Gastric lesions were induced by ethanol (70%, 0.5 mL/100 g by oral route). The animals were euthanized 1 h after the administration of ethanol and their stomachs were removed. The uninjured control group consisted of untreated animals, exposed to experimental procedures, but with no ulcer induction.

#### Quantification of non-protein sulfhydryl groups (–SH groups)

The excised stomach tissue was weighed and homogenized in a cold EDTA solution (0.02 mol/L). Aliquots of 320 μL of distilled water and 80 μL of trichloroacetic acid 50% aqueous solution were added to 400 μL of the homogenate for protein precipitation and the samples were then centrifuged at 604 × g for 15 min at 4°C. To a total of 400 μL of supernatant was added 800 μL of Tris 0.4 M (pH 8.9) and 20 μL of 5,5-dithiobis (2-nitrobenzoic acid) 0.01 M. The mixture was then stirred and the absorbance was measured at 412 nm. The concentrations of non-protein sulfhydryl groups were expressed in μg of–SH groups/g of tissue [22].

#### Determination of lipid peroxidation (LPO)

The lipid peroxidation index was determined using the method described by Ohkawa et al. [23]. The stomach tissue excised was homogenized in a cold KCl (0.15 mol/L) solution and centrifuged at 11,000 × g for 20 min at 4°C. Aliquots of 0.2 mL of sodium lauryl sulfate (8.1%), 1.5 mL of acetic acid (20%, pH 3.5), 1.5 mL of thiobarbituric acid (0.8%, w/v) and 0.3 mL of distilled water were added to 0.5 mL of the homogenate. The samples were incubated in a water bath at 95°C for 1 h. After cooling, 6 mL of an n-butanol + distilled water mixture (5:1, v/v) was added, the tubes were vortexed, and finally centrifuged at 1,073 × g for 10 min. The absorbance was measured at 532 nm and the results were expressed as μmol of MDA/g of tissue.

#### Determination of myeloperoxidase activity (MPO)

The stomach tissue excised was homogenized in 80 mM potassium phosphate buffer (PBS, pH 5,4) containing 0.5% hexadecyltrimethylammonium bromide and centrifugated at 11,000 × g for 20 min at 4°C. Aliquots of 5 μL of the supernatant were placed on the plate which was added 225 μL of a solution containing: 112.5 μL of 80 mM PBS, 95.625 μL of 0.22 mM PBS–pH 5.4 and 16.875 μL of 0.017% hydrogen peroxide. The reaction was initiated with the addition of 20 μL of 3,3´,5,5´-tetramethylbenzidine dissolved in dimethylformamide. The plate was then incubated at 37°C for 3 min and the reaction stopped by adding of 30 μL of 1.46 M sodium acetate (pH 3.0) in each well according to the method described by De Young et al. [24]. The MPO activity was determined at 620 nm and expressed as units of milli optic density (mOD)/g of tissue.

### Evaluation of healing properties of 1,8-cineole

#### Acetic acid-induced gastric ulcer

Chronic ulcer induction was based on the study of Takagi et al. [25] with some modifications. The animals were divided into three groups (n = 6/group, three females and three males), fasted for 24 h and, after this, anaesthetized for the surgical exposure of the stomach. 0.05 mL of 30% acetic acid was injected into the subserosal layer of the external wall of the stomach. One day after the surgery, daily treatment began and the animals were treated orally once a day for 14 consecutive days with 1% Tween-80 aqueous solution (control), pantoprazole (40 mg/kg) or CIN (100 mg/kg). During the treatment, the animals were observed for signs of toxicity, such as piloerection, diarrhea, changes in locomotor activity or mortality and the body weight was recorded. On the 15^th^ day, the rats were euthanized with CO_2_ gas, the stomachs were removed, photographed and the surface area of the gastric lesion were determined by computerized planimetry (Software ImageJ) and the data expressed in mm^2^.

#### Histological analyses

The stomachs were sectioned and set in 10% buffered formalin. After setting, the samples were washed with water, immersed in 70% ethyl alcohol for 3–4 days and embedded in paraffin. 5μm-thick paraffin sections were taken and stained with hematoxylin/eosin (HE) and Periodic Acid–Schiff (PAS). Histological analysis of the gastric sections was carried out using an automatic microscopy system MICRO DIP (Kacil Inc).

#### Immunohistochemical analysis

The immunohistochemical for proliferating cell nuclear antigen (PCNA), Ki-67 and bromodeoxyuridine (BrdU) was performed in 4μm-thick sections in paraffin of samples containing representative portions of the ulcerated area. Initially, the samples were deparaffinized in xylene and hydrated.

For localization of PCNA, the sections were incubated for 30 min with monoclonal antibody against the PCNA protein. Then antigenic retrieval was performed in microwave oven at 100°C. The slides were cooled to room temperature and endogenous peroxidase was blocked by the incubation in peroxidase blocking solution for 7.5 min. After cooling, the slides were incubated separately with the primary antibody for PCNA (anti-PCNA antibody [PC10]—Proliferation Marker (ab29)—Mouse monoclonal antibody, Abcan Inc, dilution 1:100, for 30 min) and with secondary antibody (Nichirei Biosciences Inc., dilution 1:200 for 30 min). The slices were, then, washed with phosphate buffered saline (PBS).

The expression of Ki-67 and BrdU proteins was detected using the free-biotin method in conjugation with HRP (Horseradish Peroxidase). The antigenic retrieval was performed by using a pressure cooker for two minutes. The slides were cooled to room temperature and endogenous peroxidase was blocked by using BSA (Bovine Serum Albumin) for 1 h. After cooling, the slides were incubated overnight with primary monoclonal anti-mouse antibody for Ki-67 protein (Santa Cruz Biotechnology, code: sc-23900, dilution 1:200) and for BrdU protein (Santa Cruz Biotechnology, IIB5, code: sc-32323, dilution 1:200). Then, it was used HRP visualization system. After washing, slides were incubated with diaminobenzidine chromogen solution (DAB), washed in water, counter-stained with hematoxylin, dehydrated and mounted. Cells immunoreactive for PCNA and for Ki-67 and BrdU were detected by the presence of a dark reddish-brown chromogen in the nucleus or nucleus/cytoplasm, respectively on epithelial cells of the lesion area. The reactivity was indicated using the following scores: positive–mild reactivity (in 10–15% of the analyzed cells), moderate reactivity (in 25–50% of the analyzed cells) and strong reactivity (in above 50% of he analyzed cells), or negative (in less of 10% analyzed cells).

### Statistical analysis

Values were expressed as mean ± standard error of mean (S.E.M). The differences between groups were determined by analysis of variance (ANOVA) followed by Tukey’s test. Statistical analysis was performed using GraphPad Prism 5.0. The level of significance for rejection of the null hypothesis was set at 5% (*p* < 0.05).

## Results

### Antiulcerogenic activity

The administration of ethanol caused extensive damage to the gastric mucosa with hemorrhagic erosions in the control group. Oral administration of CIN (50, 100 and 200 mg/kg) significantly reduced the lesion area to 40.3 ± 11.6, 4.9 ± 2.2 and 2.3 ± 1.8 mm^2^, respectively, in ethanol-induced gastric ulcer, when compared to the control group (339.8 ± 49.3 mm^2^), which corresponds to a percentage of inhibition of 88.1, 98.5 and 99.2%, respectively.

In the HCl/ethanol-induced gastric ulcer model, CIN also caused a significant level of gastroprotection (lesion areas: 28.2 ± 12.8; 11.8 ± 5.4 and 1.3 ± 0.8 mm^2^, respectively) when compared to the control group (245.5 ± 43.0 mm^2^), corresponding to 88.5, 95.2 and 99.4% of inhibition of the lesion area for doses at 50, 100 and 200 mg/kg, respectively. Pantoprazole (40 mg/kg) significantly inhibited the gastric lesions induced by ethanol and HCl/ethanol in 53.7% and 91.5%, respectively, when compared to the control group.

Subcutaneously administration of indomethacin (30 mg/kg) produced a gastric mucosal lesions index of 3.5 ± 0.5, an ulcer index of 22.8 ± 2.3 and a total index of 26.3 ± 2.7 in the control group. Pretreatment of animals with CIN at doses of 50, 100 and 200 mg/kg produced significant inhibition with all indices as shown in Table 1.

**Table 1:** Effect of oral administration of 1,8-cineole (CIN) on gastric lesions induced by indomethacin (30 mg/kg, s.c.) in Wistar rats.

Treatment	Dose (mg/kg)	Lesion index	Ulcer index	Total index
control	-	3.5 ± 0.5 (—)	22.8 ± 2.3 (—)	26.3 ± 2.7 (—)
pantoprazole	40	2.3 ± 0.4* (34.3%)	0* (100.0%)	2.3 ± 0.4* (91.2%)
CIN	50	1.2 ± 0.3* (65.7%)	9.8 ± 2.1* (57.01%)	11.0 ± 2.4* (58.2%)
	100	1.3 ± 0.4* (62.8%)	8.9 ± 4.5* (60.9%)	10.2 ± 4.7* (61.2%)
	200	0.5 ± 0.2* (85.7%)	6.3 ± 3.5* (72.3%)	6.8 ± 3.6* (74.1%)

Values represent the mean ± S.E.M. for 6 animals. The values in parentheses represent the percentage of inhibition for each parameter observed.

*Statistically different from control group, p < 0.05 (ANOVA followed by Tukey’s test).

### Effect of 1,8-cineole on gastrointestinal motility

Table 2 shows the concentration of phenol red present in the stomach and the percentage of transit intestinal of the animals treated by oral route with CIN (50, 100 and 200 mg/kg). The concentration of phenol red present in the stomach after 30 min of administration was 2.1 ± 0.9 μg/mL in animals treated with 1% Tween-80 aqueous solution (control group). The results indicate that CIN at a dose of 50 mg/kg did not affect gastric emptying, but the animals treated with the doses of 100 and 200 mg/kg showed a reduction in the rate of gastric emptying of 92.1 and 86.8% when compared to the zero time control group. The group treated with atropine (3 mg/kg, s.c), a muscarinic antagonist used as positive control, presented a reduction of 99.1% in relation to zero time control group. In the intestinal transit assay, CIN at doses of 100 and 200 mg/kg showed no effect on intestinal transit when compared to the control group. In animals treated with a dose of 50 mg/kg, the percentage of transit increased, corroborating the decrease in concentration of phenol red present in the stomach of animals treated with this dose.

**Table 2:** Effect of 1,8-cineole (CIN) on gastrointestinal motility in Wistar rats.

Treatment	Dose	Gastric emptying	Intestinal transit
	(mg/kg)	[phenol red (μg/mL)]	(%)
time 0	-	12.7 ± 0.1	-
control	-	2.1 ± 0.9	72.7 ± 3.4
atropine	3	12.6 ± 0.1	55.9 ± 5.2*
CIN	50	4.4 ± 1.7	96.0 ± 2.5*
	100	11.7 ± 0.4*	83.5 ± 2.8
	200	11.0 ± 0.7*	80.2 ± 1.9

The values indicate the concentration of phenol red retained in the stomach (gastric emptying) or the percentage of the distance traveled by the marker in relation to the total length of the small intestine (intestinal transit) 30 min after ingestion of the dye. The results represent the mean ± S.E.M. for 6 animals.

*Statistically different from control group, p < 0.05 (ANOVA followed by Tukey’s test).

### Evaluation of mucosal protective factors

#### Effect of 1,8-cineole on stimulated gastric acid secretion

After 4 h of ligation of the pylorus, it was observed that the intraduodenal administration of CIN reduced only the volume of gastric secretion. The pH and the total acidity remaining unchanged in the CIN group when compared to the control group not stimulated by secretagogues. Histamine, pentagastrin and bethanechol, when administered subcutaneously, stimulated basal gastric acid secretion increasing the volume and total acidity and decreasing the pH of gastric acid, when compared with your respective control groups. Ranitidine (60 mg/kg) and atropine (1 mg/kg) prevented the increase in volume and acidity of gastric contents, as well as decreased the pH of gastric acid secretion stimulated by histamine and bethanechol, respectively. CIN showed no inhibitory action on gastric acid secretion stimulated by histamine, bethanechol and pentagastrin (Table 3).

**Table 3:** Effect of intraduodenal administration of 1,8-cineole (CIN) on gastric secretion parameters basal or stimulated by histamine (20 mg/kg), bethanechol (2.5 mg/kg) and pentagastrin (400 μg/kg) in Wistar rats subjected to pylorus ligature.

Stimulus + treatment	Gastric volume	pH	Total acidity
	(mL)		(mEquiv. [H^+^]/mL/4 h)
control (not estimulated)	3.3 ± 0.2	2.0 ± 0.2	8.6 ± 2.2
pantoprazole (not estimulated)	2.1 ± 0.2*	3.7 ± 0.5*	3.2 ± 0.6*
CIN (not estimulated)	2.5 ± 0.2*	2.5 ± 0.1	4.8 ± 1.0
histamine	6.3 ± 0.7	1.5 ± 0.1	52.8 ± 9.9
histamine + ranitidine	2.3 ± 0.3^#^	2.6 ± 0.1^#^	2.9 ± 0.6^#^
histamine + CIN	4.6 ± 0.7	1.5 ± 0.1	36.0 ± 10.2
bethanechol	7.8 ± 0.5	1.5 ± 0.0	52.2 ± 5.4
bethanechol + atropine	2.8 ± 0.2^†^	2.5 ± 0.2^†^	6.6 ± 1.5^†^
bethanechol + CIN	6.6 ± 0.3	1.6 ± 0.0	46.8 ± 4.5
pentagastrin	7.2 ± 1.4	1.4 ± 0.0	45.0 ± 7.8
pentagastrin + CIN	4.9 ± 0.6	1.4 ± 0.0	41.0 ± 9.4

Values are expressed as mean ± S.E.M. for 6 animals. Treatment: control (1% Tween-80 aqueous solution, 0.1 mL/100 g, i.d), CIN (100 mg/kg, i.d.), ranitidine (60 mg/kg, i.d.) and atropine (1 mg/kg, s.c).

*p < 0.05 vs. control group

^#^p < 0.05 vs. histamine group and

^†^p < 0.05 vs. bethanechol group (ANOVA followed by Tukey’s test).

#### Involvement of the nitric oxide (NO) and sulfhydryl compounds (–SH groups) in gastroprotection

Both the NO synthase inhibitor, L-NAME (N_ω_-nitro-L-arginine methyl ester) and the inhibitor of sulfhydryl compounds, NEM (N-ethylmaleimide), exacerbated ethanol-induced gastric lesions in 47.8 and 148.3% respectively, compared to the groups pretreated with NaCl solution. In the animals pretreated with L-NAME, CIN continued to exert its gastroprotective effect. However, depletion of sulfhydryl groups by pretreatment with NEM, was able to significantly reduce the gastroprotective effect of CIN in relation to the group pretreated with NaCl solution (Table 4).

**Table 4:** Effect of oral administration of 1,8-cineole (CIN) on gastric lesions induced by ethanol in Wistar rats pretreated with L-NAME (N_ω_-nitro-L-arginine methyl ester, 70 mg/kg) or NEM (N-ethylmaleimide, 10 mg/kg).

Pretreatment	Treatment (p.o.)	Dose (mg/kg)	Lesion area (mm^2^)	Inhibition (%)
NaCl solution (i.p.)	control	-	298.0 ± 57.2	-
	carbenoxolone	100	42.8 ± 16.6*	85.6
	CIN	100	2.5 ± 1.2*	99.2
L-NAME (i.p.)	control	-	440.7 ± 56.98*	-
	carbenoxolone	100	43.5 ± 15.2^#^	90.1
	CIN	100	4.0 ± 2.4^#^	99.1
NEM (i.p.)	control	-	739.8 ± 110.2*	-
	carbenoxolone	100	510.2 ± 136.5	31.0
	CIN	100	709.0 ± 143.7	4.2

Results are expressed as mean ± S.E.M. for 6 animals.

*p < 0.05 compared to NaCl solution + control

^#^p < 0.05 compared to L-NAME + control (ANOVA followed by Tukey's test).

#### Effect of 1,8-cineole on the production of gastric mucus

**Fig 1:**
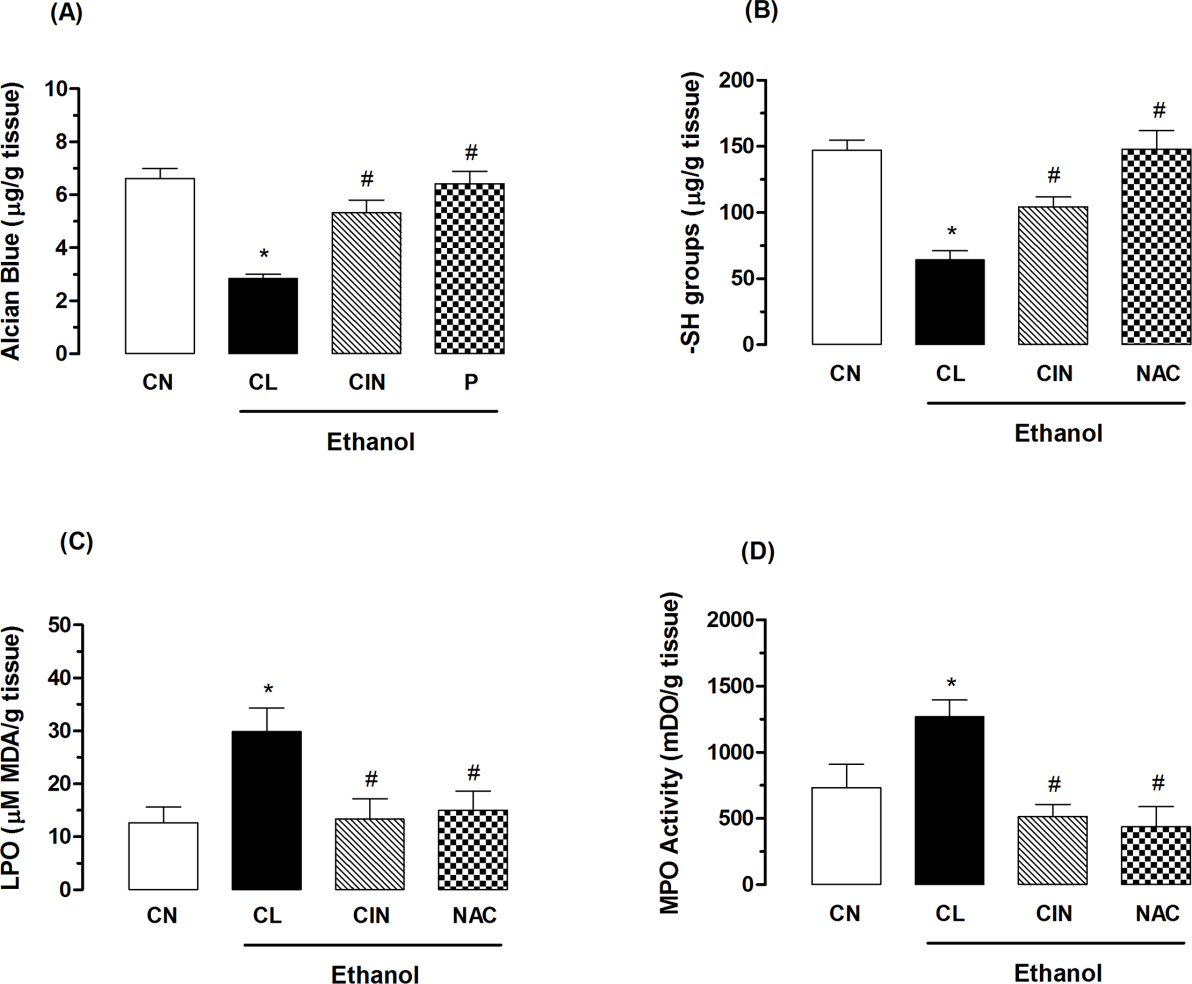
Effect of 1,8-cineole (CIN) on quantification of mucus (A), levels of sulfhydryl groups (B) levels of malondialdehyde (C) and myeloperoxidase activity (D) in the gastric ulcers model induced by ethanol. The non-injured control group (CN) received no treatment. The experimental groups received 1% Tween-80 aqueous solution (CL, injured control), pantoprazole (P, 40 mg/kg), N-acetylcysteine (NAC, 750 mg/kg) or CIN (100 mg/kg). Results are expressed as mean ± S. E. M for 6 animals. ANOVA followed by Tukey’s test (*p < 0.05 vs. non-injured control group-CN and ^#^p < 0.05 vs. injured control group-CL).

As shown in Fig 1A, the ethanol-injured control group animals showed a significant decrease in the levels of gastric mucus (2.8 ± 0.4 μg of Alcian Blue/g of tissue) compared to the non-injured control group (CN, 6.6 ± 0.4 μg of Alcian Blue/g of tissue). Treatment with CIN at a dose of 100 mg/kg was able to increase mucus production significantly in 89.3% (5.3 ± 0.5 μg of Alcian Blue/g of tissue) compared to the injured control group. Pantoprazole also caused a significant increase in the levels of gastric mucus (6.4 ± 0.5 μg of Alcian Blue/g of tissue).

#### Effects of 1,8-cineole on the antioxidant activity

The level of sulfhydryl groups (–SH groups) found in the gastric mucosa of the non-injured control group (CN) was 147.2 ± 7.5 μg/g of tissue, but in animals of ethanol-injured control group (CL) was observed a reduction in levels of GSH (64.2 ± 6.8 μg/g of tissue) compared to the non-injured control group. Oral treatment with CIN (100 mg/kg) or N-acetylcysteine (NAC, 750 mg/kg) were able to revert the reduction of the in levels of sulfhydryl groups in the gastric mucosa in 62.6 and 130.3% respectively, restoring the antioxidant system to base levels (104.4 ± 7.3 μg/g and 147.9 ± 13.8 of tissue), respectively (Fig 1B).

The lipid peroxidation (LPO) index in the gastric mucosa of rats subjected to an ethanol-induced gastric ulcer model was determined by quantification of malondialdehyde, which reacts with thiobarbituric acid. Animals in the ethanol-injured control group (CL) showed an increase in gastric levels of malondialdehyde of 136.5% (29.8 ± 4.4 μmol MDA/g of tissue) compared to the non-injured control group (CN, 12.6 ± 2.9 μmol MDA/g of tissue). Oral treatment with CIN (100 mg/kg) decreased the rate of lipid peroxidation in 55.3% by diminishing the production of malondialdehyde by ethanol (13.3 ± 3.8 MDA μmol/g of tissue). N-acetylcysteine (NAC, 750 mg/kg) also inhibited (49.6%) the increase in levels of malondialdehyde (15.0 ± 3.6 MDA μmol/g of tissue) (Fig 1C).

The myeloperoxidase activity (MPO) in non-injured control group (CN) animals was 732.1 ± 176.3 mDO/g of tissue. In the ethanol-injured control group (CL) was observed an increase in the MPO activity of 73.5% (1270.0 ± 125.6 mDO/g of tissue) when compared to the uninjured control group. Animals treated with CIN (100 mg/kg) or N-acetylcysteine (NAC, 750 mg/kg) showed a reduction in the MPO activity of 59.4% (515.0 ± 91.8 mDO/g of tissue) and 65.5% (438.0 ± 152.0 mDO/g of tissue) compared to the injured control group (Fig 1D).

### Evaluation of healing properties of 1,8-cineole

#### Acetic acid-induced gastric ulcer

**Fig 2:**
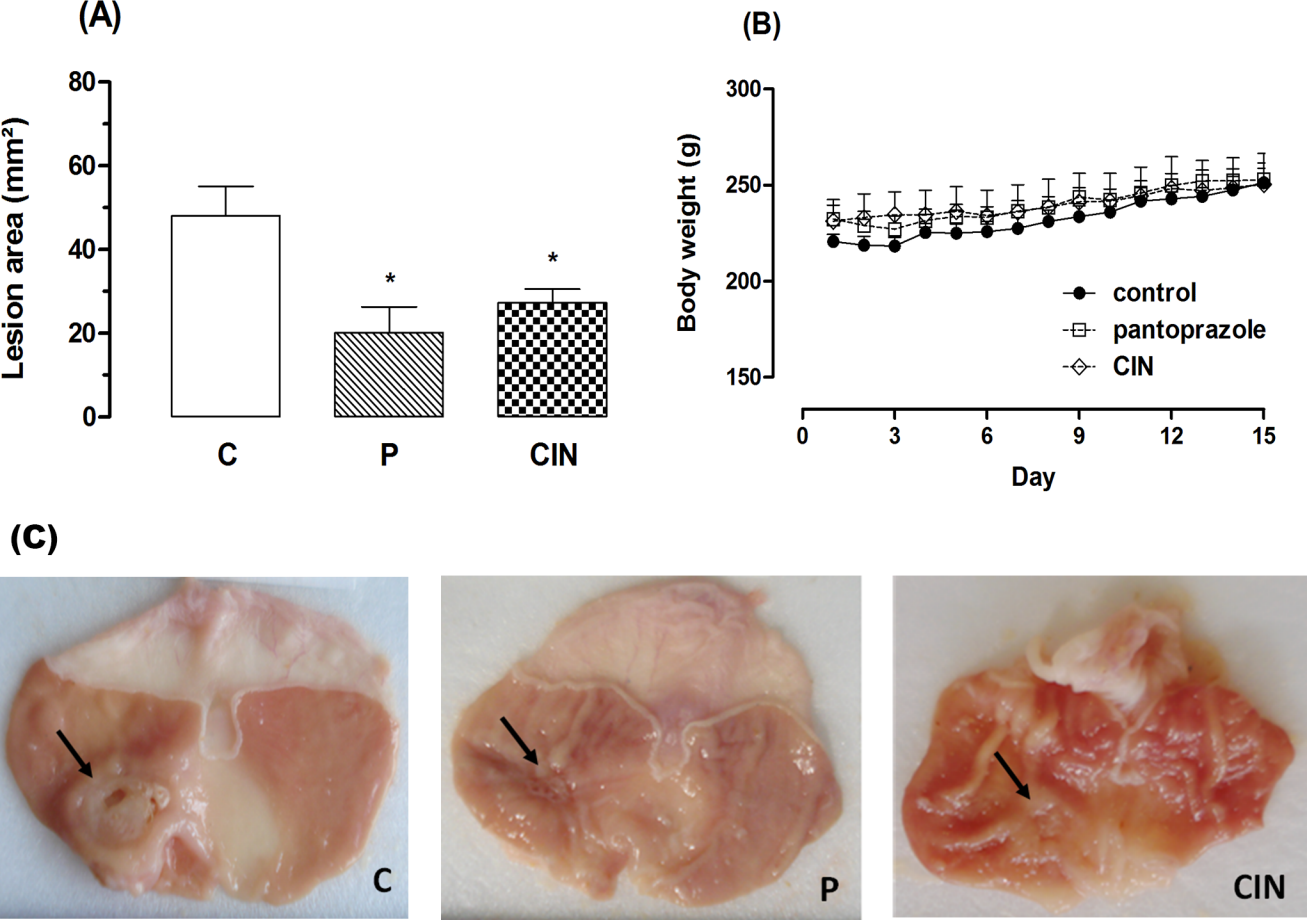
Effect of 1,8-cineole (CIN) on healing of the gastric mucosa in the rats subjected to induction of chronic ulcer by 30% acetic acid. Animals were treated orally with 1% Tween-80 aqueous solution (control group), pantoprazole (P, 40 mg/kg) or CIN (100 mg/kg) for 14 days. (A) Lesion area, (B) body weight during treatment and (C) macroscopical appearance of the gastric ulcer. Values represent the mean ± S. E. M. for 6 animals. *Statistically different from control group, ANOVA followed by Tukey's test (*p < 0.05 vs. control group).

In the acetic acid model, the results show that oral administration of CIN (100 mg/kg) for 14 consecutive days decreased (43.1%) the area of chronic ulcer to 27.3 ± 3.2 mm^2^ as can be seen in Fig 2A. Pantoprazole (40 mg/kg) speeded up the healing of gastric ulcer, significantly reducing the area of the injury to 20.1 ± 6.2 mm^2^ (58.1%), when compared to the control group (48.0 ± 7.5 mm^2^). During the 14 days of treatment, CIN or pantoprazole did not produce any visible signs of toxicity. The animals treated with CIN showed a profile of body weight gain similar to animals of control group. (Fig 2B). The macroscopic appearance of the chronic ulcer induced by acetic acid in the gastric mucosa in control and in the pantoprazole or CIN- treated groups is represented in Fig 2C.

#### Histological analyses

**Fig 3:**
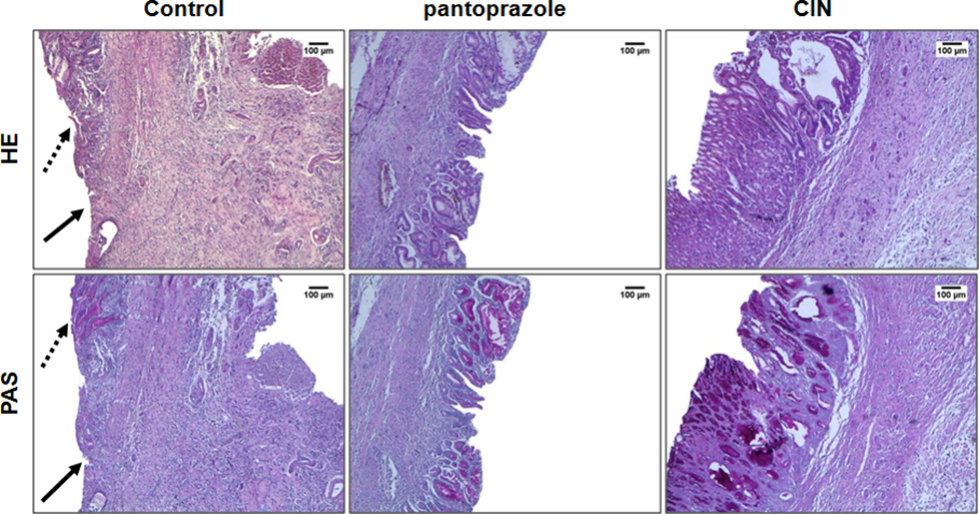
Photomicrographs of gastric mucosa stained with HE and PAS of the rats subjected to induction of chronic ulcer by 30% acetic acid. Animals were treated orally with 1% Tween-80 aqueous solution (control group), pantoprazole (40 mg/kg) or CIN (100 mg/kg) for 14 days. The filled arrow indicates the absence of the epithelial layer (ulcer area internal) and the dashed arrow indicates epithelial layer remaining (ulcer edge). Haematoxylin/eosin (HE) and Periodic Acid–Schiff staining (PAS), magnification, 40x.

Histological analysis was performed by HE and PAS staining. HE slices revealed well-defined ulcers with complete destruction of the mucosal and submucosal layer caused by acetic acid in animals of the control group. The stomachs of rats treated orally with CIN (100 mg/kg) and pantoprazole (40 mg/kg) demonstrated a regeneration of gastric mucosa, as evidenced by the reappearance of epithelial and stromal layer when compared to the control group. PAS staining also showed increased mucus production, viewed through the areas intensely stained by magenta color in the epithelial layer of mucosal (Fig 3).

#### Immunohistochemical analysis

**Fig 4:**
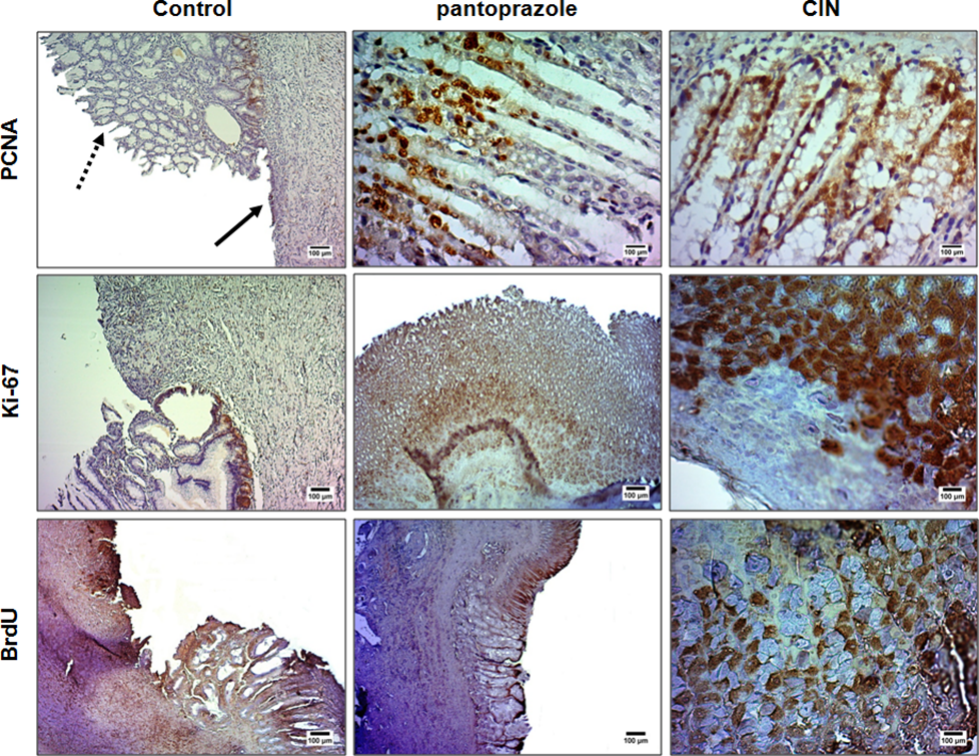
Immunohistochemical analysis for PCNA (proliferating cell nuclear antigen), Ki-67 and BrdU of the gastric mucosa of the rats subjected to induction of chronic ulcer by 30% acetic acid. Animals were treated orally with 1% Tween-80 aqueous solution (control group), pantoprazole (40 mg/kg) or CIN (100 mg/kg) for 14 days. The filled arrow indicates the absence of the epithelial layer (ulcer area internal) and the dashed arrow indicates epithelial layer remaining (ulcer edge). Microphotographs depict the immunoreactivity for PCNA, Ki-67 and BrdU in the groups, magnification 200x (control) and 500x (CIN or pantoprazole).

Immunohistochemical investigation using monoclonal antibodies against PCNA, Ki-67 and BrdU showed strong reactivity and a great quantity of PCNA- and Ki-67-positive nuclei (marked with dark reddish-brown color) and moderate reactivity for BrdU in the gastric mucosa of animals treated with CIN for 14 days, when compared to control group, in which there was no reactivity for the three markers due to the destruction of the epithelial layer, as shown in Fig 4. Treatment with pantoprazole was associated with a great quantity of PCNA-positive nuclei, moderate reactivity for Ki-67 and mild staining intensity of BrdU positive cells.

## Discussion

This study investigated the antiulcerogenic activity of monoterpene 1,8-cineole, the major compound of the essential oil of *Hyptis martiusii* (EOHM), on acute gastric lesions induced by different necrotizing agents, as well as its the mechanisms of action for the first time in the literature.

The 1,8-cineole is present in many essential oils from aromatic plants used in folk medicine and is widely used as an excipient in the pharmaceutical and cosmetics industry, for example, in nasal sprays or as a disinfectant [26] and as a food flavoring agent [27]. Many studies have reported that 1,8-cineole displays a variety of biological properties, including gastroprotective activity [9].

Our results showed that the pretreatment with CIN protected the rats’ gastric mucosa against ethanol- and acidified ethanol-induced ulcer, suggesting that the compound exhibits efficient cytoprotective activity, since it inhibits the formation of ulcerative lesions in both models. Additionally, CIN significantly reduced damage to mucosal in the indomethacin-induced gastric ulcer, a model involving NSAID, at all the doses tested, demonstrating its gastroprotective properties and suggesting the possible involvement of prostaglandins and/or mucus production in antiulcer activity. These results are in accordance with previous data described by Santos and Rao [9].

Several studies have correlated the formation of gastric ulcers with gastric emptying and gastric motility, although the data are still unclear [28]. The evaluation of the effects on gastrointestinal motility showed that higher doses of CIN administered by oral route reduced the gastric emptying rate, but did not alter intestinal transit. However, the lowest dose increased the percentage of intestinal transit, as confirmed by the decreased concentration of dye present in the stomach of animals treated with this dose. Data from the literature show that intravenous administration of 1,8-cineole delays gastric emptying and gastrointestinal transit of liquid in rats as demonstrated by Magalhães et al. [29].

The antiulcer activity of 1,8-cineole observed in the models described above led us to conduct experiments to evaluate the possible influence of mucosal protection factors, such as the antisecretory activity and the participation of nitric oxide and sulfhydryl groups on its gastroprotective effect. We verified the influence of 1,8-cineole on the parameters (volume, pH and total acidity) of basal and stimulated gastric secretion with the agonists (secretagogues) of receptors of histamine (H_2_), acetylcholine (M_3_) and gastrin (CCK_2_). The results demonstrate that CIN reduced the volume of basal acid secretion but did not decreased the total acidity as well as did not reveal any changes in the parameters for gastric acid secretion stimulated by histamine, pentagastrin and bethanechol, suggesting possible lack of antisecretory activity, different from that observed by Santos and Rao [9].

Nitric oxide (NO) and non-protein sulfhydryl groups (–SH groups) are known to be important mediators in the gastric defense mechanisms. Nitric oxide increases blood flow in the gastric mucosa, regulates mucus production and inhibits the attachment of neutrophils to endothelial cells [30]. The non-protein sulfhydryl groups (–SH groups) are directly associated with the maintenance of the integrity of the gastric mucosa, because they limit the production of free radicals and enable the production and maintenance of mucus units [31]. In animals pretreated with L-NAME, an inhibitor of NO-synthase, 1,8-cineole (100 mg/kg) continued to exert a gastroprotective effect, thereby showing that its activity does not depend on NO. In animals pretreated with NEM, an inhibitor of sulfhydryl groups, the gastroprotective effect of CIN was reduced, suggesting that the protective effect of 1,8-cineole is dependent on the presence/production of these groups.

Among the main gastric cytoprotective factors, stand out the gastric mucus, prostaglandins, adequate mucosal blood flow, nitric oxide and sulfhydryls compounds. Agents such as ethanol promote the depression of these defense mechanisms, thereby contributing to the development of lesions in the gastric mucosa [32]. Besides the previously mentioned mechanisms, the administration of ethanol is associated with increased lipid peroxidation, decreased non-protein sulfhydryl groups (–SH groups), leading to increased in the species reactive oxygen (ROS) and, additionally, it destroys epithelial cells in the stomach, leading to infiltration of inflammatory cells which eventually production of hemorrhagic lesions [33]. Confirming these data already reported in the literature, stomachs undergoing ethanol-induced damage exhibited a decrease in mucus production and levels of non-protein sulfhydryl groups (–SH groups), as well as increased levels of malondialdehyde (MDA) and myeloperoxidase activity (MPO) compared to the levels found in non-injured animals. Our results revealed a significant increase in the amount of mucus adhering to the gastric mucosa and in the basal levels of–SH groups in animals treated with 1,8-cineole (100 mg/kg), thereby explaining the gastroprotective action observed previously and the involvement of these groups in this gastroprotective effect. Pretreatment with 1,8-cineole also decreased lipid peroxidation as evidenced by reduced levels of malondialdehyde in gastric mucosa injured. The enzyme myeloperoxidase (MPO) has been reported with a marker infiltration of neutrophils in inflamed tissue, increasing their activity in several studies have been associated with the presence of peptic ulcer [34, 35]. Santos et al. [36] investigated the effects of 1,8-cineole in male Wistar rats subjected to induction of acute colitis and found that at dose of 400 mg/kg was able to reduce the MPO activity and to restore glutathione levels in the tissues of the colon, showing its potential as anti-inflammatory and preventive in gastrointestinal ulceration. In this study, 1,8-cineole (100 mg/kg, 4x a lower dose) was able to significantly reverse the increase in myeloperoxidase activity, conferring protection to the gastric mucosa by preventing neutrophil infiltration in the gastric mucosa. Taken together, these results suggest the involvement of the antioxidation mechanism of CIN in its gastroprotective effect.

This study further investigated the healing action of the compound on chronic ulcer. According to Okabe and Amagase [37], the chronic acetic acid-induced ulcer model closely resembles human ulcer peptic disease in terms of pathological features, the healing process and cycle of ulcer recurrence. The process of cell renovation of the gastric epithelium occurs every 2–4 days and the maintenance of its integrity depends on an appropriate balance between cell loss and renovation [38]. When the mucosal is damaged, or when there is the formation of an ulcer, the epithelial wound healing is started by growth and formation of gastric glands, growth of new blood vessels (angiogenesis), rapid proliferation and migration of healthy cells to injured local, leading to scar formation [39].

Our results demonstrated that, besides protecting the gastric mucosa against acute gastric lesions, 1,8-cineole also speeds up the healing of chronic ulcer acetic acid-induced. In the control group, it was observed the presence of an ulcer characterized by the partial absence of the mucosa and destruction of the submucosa. However, in the group treated with at a dose of 100 mg/kg, there was a process of re-epithelialization of the mucosa and the submucosa, evidenced by the presence of the gastric epithelial layer, as confirmed by the histological analysis (HE staining). Additionally, the same group has shown restoration of mucus production in glandular cells, demonstrating the functionality of the cells in the release of mucus that protects the mucosa, which were revealed by intense tone of pink evidenced in PAS staining.

The regenerative effect of 1,8-cineole on the gastric mucosa was assessed by detection of proliferating cells where there was the chronic ulcer. Cell proliferation can be achieved by a variety of immunohistochemical methods and antibodies, such as proliferating cell nuclear antigen (PCNA), Ki-67 and bromodeoxyuridine (BrdU), markers of proliferative activity used to evaluate the cell proliferation/DNA repair [40]. The PCNA is an auxiliary protein for DNA polymerase δ and ε, enzymes involved in DNA replication and repair, respectively. Its expression increases during the G1-phase, reaches the peaks at the S-phase, and declines during G2/M-phases of the cell cycle. This feature allows identification of the different cell cycle phases, except in cells that are out of the cell cycle [41]. The Ki-67 is a ubiquitous human nuclear protein, expressed at all stages of the cell cycle (G1, S, G2-phases and mitosis), but not in the G0-phase. Only active proliferating cells are positive for Ki-67 staining too [40]. BrdU, a synthetic nucleoside, is incorporated into nuclei during the DNA S-phase of the cell cycle. BrdU-positive cells are detected due incorporation of BrdU in the place of thymidine into replicating DNA [42]. The immunohistochemical analysis showed that there was a significant increase in cell proliferation of gastric mucosa of rats treated with 1,8-cineole for 14 days, indicating that these cells of the mucosa are in fully proliferative activity, i.e., the epithelial layer is being remade. This activity was more evident for PCNA and Ki-67. Considering that cell proliferation plays an important role in wound healing, the results observed suggests that the treatment with 1,8-cineole promoted regeneration of the gastric cells.

The acetic acid promotes an increased in volume of acid gastric secretion, mainly responsible for necrosis of mucosa and pyloric obstruction. Anti-secretory drugs (e.g. proton-pump inhibitors or H2-receptor antagonists), prostaglandin analogs (e.g. misoprostol), mucosal defense agents (e.g. sucralfate), *H*. *pylori* eradication (e.g. antibiotics) and various growth factors all significantly enhance healing of gastric ulcer including acetic acid-induced ulcers [37]. The inhibition of acid secretion significantly accelerates ulcer healing; however, antisecretory agents alone were not sufficient for improve the quality of healing, because may not suppress inflammation. Therefore, it’s necessary the combined use of antisecretory agents and gastroprotective agents for anti-ulcer treatment. [43]. It’s important to point out that the treatment with 1,8-cineole has not suppressed the gastric acid secretion, but promoted the increase in the levels of gastric mucosal protective factors (mucus and–SH groups) and the reduction in the harmful factors (lipid peroxidation and myeloperoxidase) which may be directly involved in the healing effect of the compound.

## Conclusion

Our results demonstrate, for the first time, the role of 1,8-cineole as an important ulcer healing agent and indicate that the mechanisms of action involved in this gastroprotective effect are: 1) a cytoprotective mechanism, since it causes an increase in gastric mucus; 2) antioxidant activity, preventing depletion of sulfhydryl groups and reducing levels of lipid peroxidation and myeloperoxidase activity in the gastric mucosa; and 3) healing ability. 1,8-cineole did not shown antisecretory activity. This study also provides evidence that 1,8-cineole is related to the gastroprotective effect of the essential oil of *Hyptis martiusii*.
